# Increasing African genomic data generation and sharing to resolve rare and undiagnosed diseases in Africa: a call-to-action by the H3Africa rare diseases working group

**DOI:** 10.1186/s13023-022-02391-w

**Published:** 2022-06-16

**Authors:** Aimé Lumaka, Nadia Carstens, Koenraad Devriendt, Amanda Krause, Benard Kulohoma, Judit Kumuthini, Gerrye Mubungu, John Mukisa, Melissa Nel, Timothy O. Olanrewaju, Zané Lombard, Guida Landouré, as members of the Rare Disease Working Group of the H3Africa Consortium

**Affiliations:** 1 Department of Pediatrics, Faculty of Medicine, Centre for Human Genetics, University of Kinshasa, Kinshasa, Congo; 2 Laboratoire de Génétique Humaine, GIGA-Research Institute, University of Liège, Bât. B34 +2, Sart Tilman, Avenue de l’Hôpital 13, 4000 Liège, Belgium; 3 Division of Human Genetics, National Health Laboratory Service, and School of Pathology, Faculty of Health Sciences, University of the Witwatersrand, Johannesburg, South Africa; 4 Centre for Human Genetics, University Hospital, University of Leuven, Leuven, Belgium; 5 Centre for Biotechnology and Bioinformatics, University of Nairobi, Nairobi, Kenya; 6 ADVANCE, IAVI, Nairobi, Kenya; 7 South African National Bioinformatics Institute (SANBI), University of Western Cape (UWC), Robert Sobukwe Road Bellville, Cape Town, 7535 Republic of South Africa; 8 Department of Immunology and Molecular Biology, Makerere University College of Health Sciences, Third Floor, Pathology & Microbiology building Upper Mulago Hill, P.O.Box 7072, Kampala, Uganda; 9 Neurology Research Group, Neuroscience Institute, University of Cape Town, Cape Town, 7925 South Africa; 10 Division of Nephrology, Department of Medicine, University of Ilorin and University of Ilorin Teaching Hospital, Tanke Road, PMB 1515, Ilorin, Kwara State Nigeria; 11 Julius Global Health, Julius Center for Health Sciences and Primary Care, University Medical Center Utrecht, Utrecht University, Utrecht, The Netherlands; 12 Faculté de Médecine Et d’Odontostomatologie, USTTB, Bamako, Mali; 13 Service de Neurologie, Centre Hospitalier Universitaire du Point G, Bamako, Mali

**Keywords:** Data sharing, NGS interpretation, Diversity, Reference database

## Abstract

The rich and diverse genomics of African populations is significantly underrepresented in reference and in disease-associated databases. This renders interpreting the Next Generation Sequencing (NGS) data and reaching a diagnostic more difficult in Africa and for the African diaspora. It increases chances for false positives with variants being misclassified as pathogenic due to their novelty or rarity. We can increase African genomic data by (1) making consent for sharing aggregate frequency data an essential component of research toolkit; (2) encouraging investigators with African data to share available data through public resources such as gnomAD, AVGD, ClinVar, DECIPHER and to use MatchMaker Exchange; (3) educating African research participants on the meaning and value of sharing aggregate frequency data; and (4) increasing funding to scale-up the production of African genomic data that will be more representative of the geographical and ethno-linguistic variation on the continent. The RDWG of H3Africa is hereby calling to action because this underrepresentation accentuates the health disparities. Applying the NGS to shorten the diagnostic odyssey or to guide therapeutic options for rare diseases will fully work for Africans only when public repositories include sufficient data from African subjects.

## Context

On February 28th 2022 the world celebrated the 14th Rare Diseases Day, and then the sixth Undiagnosed Diseases Day was celebrated on April 29th. These and previous such days have been opportunities for the members of the Rare Disease Working Group of the Human Heredity and Health in Africa Consortium (H3Africa) to reflect on major barriers that cause a high proportion of patients with rare diseases in Africa to remain undiagnosed. The Rare Disease Working Group is comprised of delegates from H3Africa-funded projects that work within the rare genetic disorder niche. Their research initiatives are aimed at identifying and filling the knowledge gaps in rare disease in Africa by characterizing the clinical and molecular epidemiology of specific groups of rare diseases, including developmental delay, deafness, neurodegenerative and neuromuscular diseases. This group meets monthly to explore barriers to the implementation of genomic medicine for rare diseases and to engage with relevant stakeholders to promote rare genomic disease research in Africa.

About 3.5–5.9% of the world population are affected by a rare disease [1]. This corresponds to around 50 million people in Africa and represents a large community of individuals and families in need of diagnostics and care. About 72% of rare diseases are thought to have a genetic etiology [1]. The completion of the Human Genome Project in April 2003 offered the opportunity to translate this unprecedented scientific accomplishment into tangible diagnostic improvements in human health [2], especially for rare and undiagnosed disease patients. Next Generation Sequencing (NGS) technology has facilitated major diagnostic progress triggered by the availability of a human reference genome. This tool has significantly accelerated the pace of disease gene and therapeutic discovery for rare diseases [3]. Moreover, NGS is shortening the diagnostic odyssey by its ability to explore multiple types of genomic aberrations at once, even without a clear clinical hypothesis. This has led to the implementation of rapid NGS testing for critically ill patients in Europe [4], America [5, 6], Australia [7] and the United Kingdom [8], with a turn-around-time that allows NGS-guided therapeutic adjustments to be made.

In Africa, there is a sharp contrast between the implementation of NGS in pathogen genetics versus in human genetics. Multiple financial international supports are available for technology transfer to Africa for pathogen genetics. The Ebola epidemic in West Africa and the Covid-19 response have created a consensus on the utility of NGS on the continent and offered a momentum for broad expansion of short and long reads sequencing technologies as routine testing of pathogens in Africa [9, 10]. With regards to rare diseases, genetic service delivery in general, and NGS-based testing in particular, remains limited and extremely fragile in Africa. NGS-based diagnostics tests are not offered routinely to rare diseases patients in Africa yet. The main barriers include limited existing infrastructure and processes, insufficient funding and lack of political support, and poorly structured health systems [11]. However, research projects such as the Deciphering Developmental Disorders in Africa (DDD-Africa, South Africa and the Democratic Republic of Congo), Hearing Impairment Genetics Studies in Africa (HI-GENES Africa, South Africa, Cameroun, Ghana, Mali), Clinical and Genetic Studies of Hereditary Neurological Disorders in Mali or the genetic study of neuromuscular diseases (South Africa) are ensuring rare diseases patients in Africa can receive NGS tests. In addition, there is an important contribution of NGS tests free-of-charge to African rare diseases patients from international research collaborations and philanthropic initiatives such as the Centers for Mendelian Genomics (CMG) [12] and the iHope foundation. All these efforts have allowed ending diagnostic odyssey in many African patients and adjusting care when possible as well as identifying new disease genes [13–18].

Generating genomic data is only the first step on the path toward resolving and managing undiagnosed genetic diseases. The second and most important part for patients is interpreting those data with regards to their health conditions and potential management. For unaffected parents and relatives, a diagnosis answers questions around recurrence risk and offers opportunities for prenatal testing, where relevant. While allowing a broader exploration of the genome, new sequencing technologies identify an enormous amount of genomic variation, causing increased complexity in data interpretation [19]. Correlating the molecular findings with a clinical phenotype contributes to overcoming this issue. In depth clinical phenotyping therefore provides critical morphological information for clinical variant interpretation [20]. However, multiple rare diseases have atypical facial presentations or remain uncharacterized in understudied populations. At the end, many patients with rare diseases remain undiagnosed despite access to high resolution sequencing technologies. Over the past years, the H3Africa has been contacted by multiple international clinical geneticists and researchers seeking to access African data in order to gain a better understanding of candidate variants identified by their laboratories in patients with undiagnosed rare developmental diseases. Therefore, the limited ability to fully interpret genomic data is contributing to a high burden of undiagnosed diseases, and represents a bottleneck shared by all patients irrespective of their background and geographic location.

This manuscript presents the view of the H3A-RDWG for the improvement of the resolution of rare and undiagnosed diseases.

## Challenges facing Africa for resolution of rare undiagnosed diseases

Clinical interpretation of genomic data for rare diseases is complex and challenging. This process consists of filtering variants based on knowledge gathered from different sources (disease databases and literature, population databases labelled as reference databases, existing functional data), phenotypic overlap, segregation data (inheritance) and computational predictions (in silico tools). Variants are then prioritized based on different criteria which favor a pathogenic or benign interpretation, and finally classified into five categories according to the guidelines from the American College of Medical Genetics and Genomics and the Association for Molecular Pathology (ACMG-AMP) [21]. Considering that functional data are not available for the vast majority of variants, and that the phenotypic spectrum of many rare undiagnosed diseases is not known or is poorly characterized in understudied communities, the population frequency remains among the strongest filters for NGS data interpretation. Therefore, the lack of representation in population frequency databases makes clinical interpretation of genomic data significantly more challenging in Africa and in African diaspora. In 2021, the H3Africa was contacted by a team in Canada, seeking information regarding 3 genomic variants identified in patients of African origin with rare diseases in Canada. The query in the H3Africa reference panel and in the internal database of the Center for Human Genetics of the University of Kinshasa indicated that two of these variant were absent and the last had very low frequency with no homozygous (AF = 0.0078). We were able to provide further evidence of the novelty or the rarity of these variants in an African dataset. This illustrate the complexity of data interpretation even in developed countries and supports the value of data sharing.

The concept of underrepresentation encompasses two components, first the paucity of samples from African individuals, second the failure to capture the African granularity. The African continent is recognized for its great genomic diversity. Underneath this great continental diversity, Choudhury et al., (2020) [22] reported large differential allele frequencies between and within African populations, which supports previous indications that geographical and ethno-linguistic lineages broadly define genetic relationships in sub-Saharan Africa [23, 24]. Therefore, it is plausible that historical, cultural and geographical contexts may have created multiple genetic sub-communities or clusters within Africa, thus resulting in a fine granularity within the African continent. Because of such granularity, variants as well as some undiagnosed diseases may possibly be restricted to one or a few particular African clusters. Although multiple efforts are being undertaken to increase the volume of African samples, the great African diversity and the fine granularity could have prevented an even inclusion of samples from people of African ancestry [25].

The first consequence of the underrepresentation of African samples from commonly used databases is the higher level of difficulty to reach a molecular diagnosis in African individuals. The data disparity ultimately results in health disparity [17]. There is a risk that NGS will be perceived as deeply flawed and thus of limited clinical utility for some populations, which would work against all of the efforts to make this technology more accessible on the African continent. The call for global action for rare diseases in Africa that followed the 11^th^ International Conference on Rare Diseases and Orphan Drugs (ICORD) emphasized that the chance of a false-positive result is greater in populations with a paucity of genetic reference data [26]. Another consequence is the misclassification of non-pathogenic variants in existing disease-related databases. Many variants are being inaccurately classified as pathogenic or likely pathogenic because of their novelty or rarity in reference databases [27], compounded by the lack of diversified frequency data. As previously demonstrated, filtering the most reputable disease database, ClinVar (https://www.ncbi.nlm.nih.gov/clinvar/), against a good population database illustrated enrichment of ClinVar with a significant proportion of wrongly ascertained variants [27]. Also, re-analysis of NGS data after databases have been populated helped in both identifying previously discarded plausible variants and down-classifying previously misclassified variants [28–31]. Such misclassification was found to be higher in individuals of African ancestry [32]. The enrichment of reference databases is recognized to play a key role in the improvement of NGS-based diagnostics [28].

## Opportunities from Africa

Simulations showed that increasing the number of samples from African ancestry individuals in reference databases would significantly prevent misclassification of variants [22, 32]. Therefore, increasing diversity in databases with diverse African data will be a powerful tool to improve the diagnostic yield and the diagnostic accuracy for African and non-African rare undiagnosed diseases patients. Fortunately, the production of genomic data from African individuals has significantly increased over the last decade. This production has been significantly facilitated by the H3Africa Consortium, funded by the National Institute of Health (NIH) and Wellcome Trust foundation [33]. Although most of the funded projects focused on complex and infectious diseases, research participants in those projects would qualify to populate an aggregate reference frequency database for rare diseases. An aggregate reference frequency populated with African data will be a game changer in resolving undiagnosed diseases not only in Africa but for 3.5–5.9% of the world population. A good illustration of the power of African data is the recent submission by the H3Africa of 41 variants to the ClinVar (https://www.ncbi.nlm.nih.gov/clinvar/). Some of these variant had conflicting interpretation in ClinVar but were all observed in more than 5% of the H3Africa participants. Adding this information from H3Africa data allowed these variants to be reclassified as benign.

Another initiative poised to have a significant impact is the Africa Pathogen Genomics Initiative (PGI) developed by the Africa CDC Institute for Pathogen Genomics, which aims to build and host an African-owned data library and real-time data sharing platform, as well as to expand and strengthen laboratory and bioinformatics capacity.

## The call-to-action

It is clear that African researchers must join forces to address the paucity of African genomic data in the public domain, its impact on the diagnostic yield and accuracy for rare undiagnosed disease patients, and promote data sharing. The Genome Aggregation Database (gnomAD) consortium with its aggregate reference frequency browser (https://gnomad.broadinstitute.org) is recognized as the most reputable source for reference frequency in the interpretation of variants with regards to rare diseases. We call here for investigators who have generated genomic data from African participants to help the rare and undiagnosed diseases community by sharing the data through the gnomAD effort. Thus far, the high sensitivity of African data is often identified as the main limit to sharing African data in gnomAD. Other investigators report the lack of proper consent for large scale sharing of data. Although the legitimacy may be discussed and should be addressed going forward, the issue with sharing preexisting African genomic data requires customized approach. Such customization is being implemented in the African Genome Variation Database (AGVD, https://agvd-dev.h3abionet.org/). The AGVD, developed by the pan-African bioinformatics network for H3Africa (H3A-Bionet) [34], will contain creative solutions with multiple layers of controlled access for sharing aggregate frequencies from African research participants, including the more sensitive data. The AGVD will also host a beacon for data collected in rare diseases research through the African Rare Diseases Initiative (ARDI). We also call the patients and advocacy groups as well as ethics committees to be more active in the discussion about the consent for sharing of aggregate summary data.

Existing H3Africa data contain multiple gaps that prevent the full diversity and fine granularity of the African genome from being captured [22]. An estimated three million African genomes will be needed to reach the expected diversity in reference databases [35, 36]. A strong commitment from funders is needed to work with African researchers to reach this goal.

Therefore, we call on local and international funding agencies to support the production of more African genomic data. Funding strategies that will enable smaller, locally relevant genomics studies as well as large-scale precision genomics initiatives and genomic data science are needed to comprehensively address African genomic data shortages and apply the findings in improving health in all regions of the world. International collaborative research grants that bring together a diverse group of researchers from different countries, continents and research niches will be particularly useful to complement and address skills- and experience gaps. In addition to calling the NIH and the Wellcome Trust to expanding their funding, we are calling for the European Union to consider data diversity as a major challenge to be addressed by the Beyond One Million Genomes Project. We believe that the African-Japan collaborative research, the China Africa Research Initiative (SAIS-CARI) and the Australian’s Partnership & Research Development Fund (PRDF) should specifically consider increasing African genomic data as one of their transversal theme.

While emphasizing the usefulness of sharing data collected from reference individuals, it is worth noting that sharing deidentified patients variant is also needed. Beside increasing the diversity in disease-associated databases, such submissions also help data contributors in improving the diagnostic in their own patients. For instance, sharing variants through ClinVar, DECIPHER and MatchMaker Exchange (MME) will also provide the submitter with additional evidence for better classification of their variants. The “follow” button in ClinVar keeps the submitter and other users informed about any change occurring in the classification of their variant of interest. The DDD-Africa research project and the Center for Human Genetics of the University of Kinshasa are already taking advantage of the DECIPHER and MacthMaker Exchange to increase their diagnostic yield for African patients with developmental disorders and intellectual disability. More African projects, laboratories and clinics are invited to use those resources for the ultimate benefits of African patients.

In summary, to be able to respond to the challenge of improving the resolution and care for undiagnosed rare diseases, there is a need to address data disparity by (1) making consent for sharing aggregate frequency data an essential component of research toolkit; (2) encouraging investigators with African data to share available data through public resources such as gnomAD, AVGD, ClinVar, DECIPHER and to use MatchMaker Exchange; (3) educating African research participants on the meaning and value of sharing aggregate frequency data; and (4) increasing funding to scale-up the production of African genomic data that will be more representative of the geographical and ethno-linguistic variation on the continent. This would ultimately lead to improved diagnosis and management of patients with rare disease both on the African continent and in the rest of the world.

## Data Availability

Data sharing not applicable to this article as no datasets were generated or analyzed during the current study.
